# Gene expression analysis in endometriosis: Immunopathology insights, transcription factors and therapeutic targets

**DOI:** 10.3389/fimmu.2022.1037504

**Published:** 2022-11-30

**Authors:** Rong Geng, Xiaobin Huang, Linxi Li, Xin Guo, Qingru Wang, Yuhua Zheng, Xiaoling Guo

**Affiliations:** ^1^ Department of Gynecology, Affiliated Foshan Maternity & Child Healthcare Hospital, Southern Medical University, Foshan, China; ^2^ Department of gynecology, The Second School of Clinical Medicine, Southern Medical University, Guangzhou, China

**Keywords:** endometriosis, immune disorder, transcription factors, ceRNA network, goserelin and dienogest

## Abstract

**Background:**

Endometriosis is recognized as an estrogen-dependent inflammation disorder, estimated to affect 8%-15% of women of childbearing age. Currently, the etiology and pathogenesis of endometriosis are not completely clear. Underlying mechanism for endometriosis is still under debate and needs further exploration. The involvement of transcription factors and immune mediations may be involved in the pathophysiological process of endometriosis, but the specific mechanism remains to be explored. This study aims to investigate the underlying molecular mechanisms in endometriosis.

**Methods:**

The gene expression profile of endometriosis was obtained from the gene expression omnibus (GEO) database. Gene set variation analysis (GSVA) and gene set enrichment analysis (GSEA) were applied to the endometriosis GSE7305 datasets. Cibersort and MCP-counter were used to explore the immune response gene sets, immune response pathway, and immune environment. Differentially expressed genes (DEGs) were identified and screened. Common biological pathways were being investigated using the kyoto encyclopedia of genes and genomes (KEGG) pathway enrichment analysis. Transcription factors were from The Human Transcription Factors. The least absolute shrinkage and selection operator (Lasso) model identified four differential expressions of transcription factors (AEBP1, HOXB6, KLF2, and RORB). Their diagnostic value was calculated by receiver operating characteristic (ROC) curve analysis and validated in the validation cohort (GSE11691, GSE23339). By constructing the interaction network of crucial transcription factors, weighted gene coexpression network analysis (WGCNA) was used to search for key module genes. Metascape was used for enrichment analysis of essential module genes and obtained HOXB6, KLF2. The HOXB6 and KLF2 were further verified as the only two intersection genes according to Support Vector Machine Recursive Feature Elimination (SVM-RFE) and random forest models. We constructed ceRNA (lncRNA-miRNA-mRNA) networks with four potential transcription factors. Finally, we performed molecular docking for goserelin and dienogest with four transcription factors (AEBP1, HOXB6, KLF2, and RORB) to screen potential drug targets.

**Results:**

Immune and metabolic pathways were enriched in GSVA and GSEA. In single sample gene set enrichment analysis (ssGSEA), most immune infiltrating cells, immune response gene sets, and immune response pathways are differentially expressed between endometriosis and non-endometriosis. Twenty-seven transcription factors were screened from differentially expressed genes. Most of the twenty-seven transcription factors were correlated with immune infiltrating cells, immune response gene sets and immune response pathways. Furthermore, Adipocyte enhancer binding protein 1 (AEBP1), Homeobox B6 (HOXB6), Kruppel Like Factor 2 (KLF2) and RAR Related Orphan Receptor B (RORB) were selected out from twenty-seven transcription factors. ROC analysis showed that the four genes had a high diagnostic value for endometriosis. In addition, KLF2 and HOXB6 were found to play particularly important roles in multiple modules (String, WGCNA, SVM-RFE, random forest) on the gene interaction network. Using the ceRNA network, we found that NEAT1 may regulate the expressions of AEBP1, HOXB6 and RORB, while X Inactive Specific Transcript (XIST) may control the expressions of HOXB6, RORB and KLF2. Finally, we found that goserelin and dienogest may be potential drugs to regulate AEBP1, HOXB6, KLF2 and RORB through molecular docking.

**Conclusions:**

AEBP1, HOXB6, KLF2, and RORB may be potential biomarkers for endometriosis. Two of them, KLF2 and HOXB6, are critical molecules in the gene interaction network of endometriosis. Discovered by molecular docking, AEBP1, HOXB6, KLF2, and RORB are targets for goserelin and dienogest.

## Introduction

Endometriosis is one of the most common reproductive disorders in which functioning endometrial glands and stroma are aberrantly located outside the uterine cavity. Those with the condition often suffer from dysmenorrhea, dyspareunia, infertility, and pelvic pain, which negatively impact patients’ quality of life. Endometriosis is a benign disease. However, its biological characteristics are similar to tumors, such as estrogen dependence, progressive and invasive growth, recurrence, and a tendency to metastases (1, 2). It carries an increased risk of developing ovarian cancer, particularly clear cell carcinomas and ovarian endometrioid carcinomas (3). The pathogenesis of endometriosis is thought to be multifaceted. So far, the most widely accepted theory is retrograde menstruation (4). In addition, coelomogenesis, genetic factors, immune system disorders, environmental factors, and lifestyle changes contribute to the development of endometriosis. Recently, multiple types of omics data were applied to research on endometriosis. A large number of genes were identified in the occurrence of endometriosis, affecting immune system regulation, cell adhesion, vascularization, and more. However, non-invasive approaches in diagnosis and treatment remain to be addressed. Therefore, enhanced understanding of the pathogenesis of endometriosis should therefore offer candidates for diagnostic and therapeutic targets.

As an estrogen-dependent chronic inflammatory disorder, the immune–hormonal loop is believed to play critical role in the etiology of endometriosis. Immune dysfunction facilitates endometrial debris successful development after implanting into ectopic locations. Disrupting immune homeostasis induces secretion of growth factors and cytokines that promote the survival of ectopic endometrial cells (5, 6). The immune cell population, including neutrophils, macrophages, dendritic cells (DCs), natural killer (NK) cells, T helper cells and B cells, are maladjusted (7–12). In addition, cytokines and chemokines involved in inflammation, angiogenesis, and tissue growth are increased in plasma and peritoneal fluid (13, 14).

Transcription factors (TFs) directly interpret the genome and usually perform the first step in decoding DNA sequences. Many genes act as’ master regulators’ and ‘selection genes,’ exerting control over processes including cell types, developmental patterns (15) and specific biological pathways, such as immune responses (16). Aberrant TFs, involved in abnormal biologic consequences in endometriosis, including estrogen excess, immune inflammation, and sprouting angiogenesis, may represent a class of therapeutic targets. This led us to speculate a large number of TFs in endometriosis. Adipocyte enhancer binding protein 1 (AEBP1) is linked to adipogenesis and smooth muscle cell differentiation. It is found that abnormal expression of AEBP1 may be closely related to the occurrence of various tumors. In glioblastoma, AEBP1 was negatively correlated with cluster of differentiation CD8+ T cells and purity, and it seemed positively associated with CD4+ T cells, B cells andDCs (17). AEBP1 has also been found to have clinical significance and biological function in colonic adenocarcinoma . Its overexpress in colonic adenocarcinoma tissues indicated a poor prognosis. In addition, its silencing inhibited the proliferation, migration, and invasion ofcolonic adenocarcinoma cells (18). Homeobox B6 (HOXB6) is thought to be involved in some acute myeloid leukemia and colorectal cancer. It acts as a transcription factor by forming synergistic DNA-binding complexes with PBX or other protein partners. HOXB6 signal is a cytoplasmic signal throughout fetal epidermal development, but is mainly the nuclear signal in normal adult skin (2, 19). Kruppel Like Factor 2 (KLF2) participates in a wide range of physiological and pathological processes, for instance, adipogenesis, embryonic erythropoiesis, epithelial integrity, inflammation, and T-cell motility (20, 21). KLF2 deficient mice manifested aberrant angiogenesis, resulting in fetal bleeding and death. KLF2 was also capable of blocking endothelial cell apoptosis, promoting metabolic stasis, and reducing metabolic dependence on glucose (22). RAR Related Orphan Receptor B (RORB) is mainly expressed in the central nervous system, retina, and pineal gland and is also differentially expressed in bone, pancreas, and endometrial carcinoma tissues (23, 24). Human islets express a high level of RORB (25), and RORB can suppress the Wingless/int1 (WNT) pathway by enhancing the expression of HBP1, a member of high mobility group (HMG) transcription factor (26). Researches on the above four molecules in endometriosis remain limited.

Using gene expression omnibus (GEO) download gene expression profile data (27) and data enrichment, we found a certain correlation between endometriosis and systemic lupus erythematosus (SLE), complement, coagulation cascades, and metabolity-related pathways. We then screened for immune infiltrating cells, immune response gene sets, and immune response pathways closely associated with endometriosis. Furthermore, we demonstrated the differentially expressed genes (DEGs) and found an intersection between DEGs and TFs. We assess the diagnostic value of selected five TFs from the lasso model using the receiver operating characteristic (ROC) curve calculation. We also confirmed the results in the validation queue. Then we constructed an interaction network for the remaining four key TFs (AEBP1, HOXB6, KLF2, and RORB) and screened key modules by weighted gene coexpression network analysis (WGCNA). Sixty-two genes in models regained from WGCNA were all put into Metascape, and KLF2 and HOXB6 genes were again present in modules based on Metascape. Using other machine learning approaches, both KLF2 and HOXB6 repeatedly appeared in Support Vector Machine Recursive Feature Elimination (SVM-RFE) and random forest models, implying their crucial role in endometriosis. LncRNA-miRNA-mRNA network was established to assess regulatory relationships for AEBP1, HOXB6, RORB, and KLF2. Finally, for the above four protein structures, we used autotool software to predict goserelin and dienogest binding sites. The results emphasized above four genes are annotated as the targets of goserelin and dienogest.

## Materials and methods

### Enrichment analysis of gene set variation analysis and gene set enrichment analysis in endometriosis

GSVA and GSEA assess enrichment in endometriosis. GSVA is a nonparametric, unsupervised analysis method to determine gene enrichment in the transcriptome. GSVA evaluates the enrichment of metabolic pathways across samples by transforming genes into a gene set expression matrix across samples. GSEA estimates the distribution trend of genes in a pre-defined gene set in a gene table ranked by phenotype correlation, thereby judging their contribution to phenotype. Gene enrichment of endometriosis was assessed by GSVA and GSEA of the Sangerbox tools (28). The Sangerbox tool is a free online data analysis platform.

### Microarray data collection obtained from the GEO database

Use the GEO query package of R software (version 4.1.2) to obtain the data set GSE7305 (n=10 endometriosis, n=10 normal endometrium) as the test set and get the data set GSE11691 (n=9 endometriosis, n=9 normal endometrium), data set GSE23339 (n=10 endometriosis, n=9 normal endometrium) as the validation set. All data sets are normalized. All data sets are standardized through the Sangerbox tools (28).

### Evaluation of immune cell infiltration and immune landscape in endometriosis

We evaluated immune-infiltrating cells by Cibersort and MCP-counter, respectively, and evaluated the immune response gene set and immune response pathway in endometriosis by single sample gene set enrichment analysis (ssGSEA) through the Sangerbox tools (28). The proportion of infiltrated immune cells was shown in the histogram drawn by the multi-group stacked bar graph drawing tool. Matrix correlation analysis and visualized mapping of immune infiltrating cell correlation heatmap. The “ggplot2” package of R software (version 3.6.3) was used to create differential maps showing immune cell infiltration, immune response genomes, and differential expression of immune response pathways. The “ggstatsplot” package of R software (version 3.6.3) was used to visualize 27 differentially expressed transcription factors and their association with immune infiltrating cells, immune response gene sets, and immune response pathways. P values ​​< 0.05 were considered statistically significant. The immune response gene sets and immune response pathways data were from GSEA (29).

### DEGs acquisition and enrichment analysis in GSE7305 and GSE11691 datasets

Through Sangerbox tools, GSE7305 DEGs and GSE11691 data set were identified, the log a fold change |log2FC| > 1, P values < 0.05, DEGs were defined as statistically significant. Volcano maps and heat maps were used to visualize these DEGs better. Enriched DEGs analysis was performed using Gene Ontology (GO) and Kyoto Encyclopedia of Genes and Genomes (KEGG) paths, through the “GOplot” package and “ggplot2” of R software (version 3.6.3) and visualized with circle and chord plots.

### Differentially expressed transcription factors identified in endometriosis

Human transcription factor data were downloaded from the human transcription factor database (30). By overlapping transcription factors and common DEGs, differentially expressed TFs were identified for further analysis.

### The least absolute shrinkage and selection operator regression model was used to analyze the differentially expressed TFs

The Lasso regression model is fitted with a generalized linear model with variable screening and complexity adjustment. Therefore, regardless of whether the target dependent variable is continuous, or binary or multivariate discrete, it can be modeled and predicted by Lasso regression. We performed a Lasso analysis of twenty-seven differentially expressed transcription factors by the Sangerbox tools (28) to obtain a model with fewer genes.

### Identification, validation, and ROC curve for diagnostic markers

Expression levels and ROC analysis of key genes were identified in GSE7305 using “ggplot2” and “pROC” of R software (version 3.6.3). Expression level and ROC analysis of genes identified in endometriosis (GSE7305) using the validation set GSE11691. P values < 0.05 were considered statistically significant. In general, the area under the curve (AUC)>0.9 represents a high diagnostic value, 0.7<AUC ≤ 0.9 represents moderate diagnostic value, and 0.5<AUC ≤ 0.7 represents a low diagnostic value.

### The interaction network of AEBP1, HOXB6, KLF2 and RORB constructed by the string database

The String is a database that can be used to retrieve interactions between known proteins and predictive proteins. Through the String database, we predict the respective interaction network of four key transcription factors (AEBP1, HOXB6, KLF2, and RORB).

### WGCNA analysis of the interaction network of AEBP1, HOXB6, KLF2, and RORB

The WGCNA is a method of a genetic set with similar identification mode for four essential genes interaction networks. By analyzing the connection between genes and sample phenotypes, the regulation network between genes is drawn, and the key regulation is appraised gene. ImageGP (31) is a free online map platform and provides WGCNA analysis to find AEBP1, HOXB6, KLF2 and RORB interaction networks related to clinical properties. The Pearson-related analysis is performed between genetic pairs, and the results are used to build a corresponding Pearson-related analysis. Two or three module genes with Gausson-related values (Pearson-related values > 0.6).

### Metascape analysis of the interaction network of four genes

Metascape (32) is a powerful gene function annotation tool to realize gene or protein function. Metascape enriched the key module genes obtained in WGCNA. Among 85 genes, 62 were included in the Metascape enrichment analysis.

### Support vector machine recursive feature elimination for genes selection

SVM-RFE algorithm was used to screen new feature genes (33). The SVM-RFE model builds a ranking weight vector caused by SVM training, which removes smallest ranking attribution in each iteration, and obtained a ranking the weight vector (34)

### Random forest classification model

The algorithm of random forest classification was according to the multiple decision trees. The number of trees in the forest was set 500. Feature importance was determined by the Mean Decrease Gini Index calculated by the random forest (35). We used random forest to construct a predictive model to evaluate importance of variable TFs according their contribution to the group accuracy (34).

### Construction of LncRNA-mRNA-miRNA network

StarBase (36), DIANA-microT (37), Miwalk (38), MiRDB (39), and TargetScan (40) were used to judge interactions between lncRNAs, miRNAs and target mRNAs. Constructing a lncRNA-miRNA-mRNA ceRNA network by Sangerbox tools (28).

### Protein-ligand interaction analysis

Autodock is an open-source molecular simulation software designed to perform interactions between small molecule ligands and macromolecule receptors for drug design, discovery, and virtual screening. To predict the binding conformation and binding free energy of goserelin or dienogest ligands to transcription factors, amino acid sequences of transcription factors were first obtained from the UniProt database (41). In AlphaFold Protein Structure Database (42), protein structure prediction of transcription factors as the receptor, the 2d structure of small molecule downloaded from https://pubchem.ncbi.nlm.nih.gov/, as the ligand. Finally, the binding of receptor and ligand was predicted by Autodock (4.2.6) and MGLTools (1.5.7). Pymol (4.6.0) was used to remove the structure of solvent before molecular docking. Docking pockets with binding energies below -1.2 kcal/mol are considered good binding energy conformations.

## Results

### GSVA and GSEA analysis of KEGG

**Figure 1:**
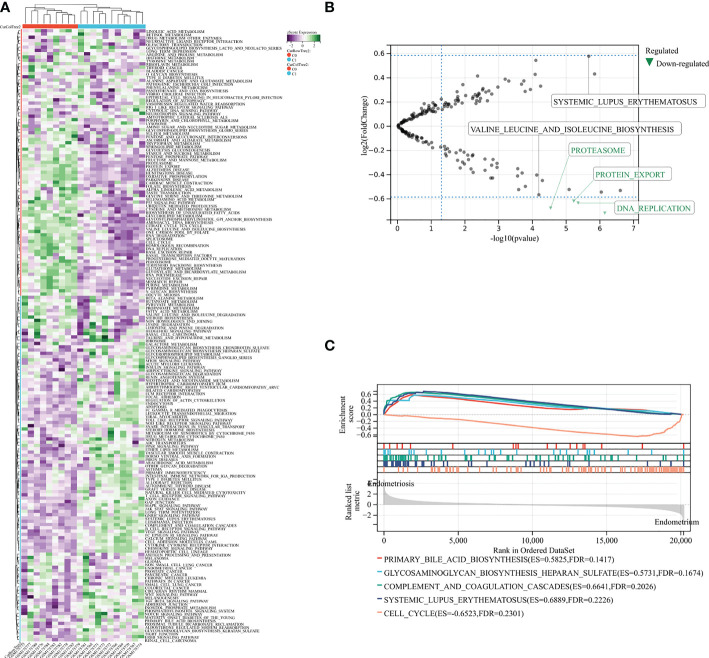
GSVA and GSEA analysis in exploration GSE7305 dataset for endometriosis. **(A)** The pathway enrichment by GSVA. **(B)** Selected top five pathways from GSVA enrichment. **(C)** Main pathways analysis by GSEA. C0: normal endometrium. C1: endometriosis.

To examine pathway alterations in endometriosis, we performed GSVA to study GSE7305 dataset (**Figure 1A**). The volcano diagram revealed the top five enrichment pathways (**Figure 1B**), namely, SLE, valine leucine and isoleucine biosynthesis, proteasome, protein export, DNA replication. Pathway enrichment from GSEA was summarized in **Figure 1C**, such as primary bile acid biosynthesis, glycosaminoglycan biosynthesis heparan sulfate, complement and coagulation cascades, SLE, and cell cycle. The above results imply that endometriosis correlates with immune and metabolic aspects.

### Cibersort for immune infiltrating cells

**Figure 2:**
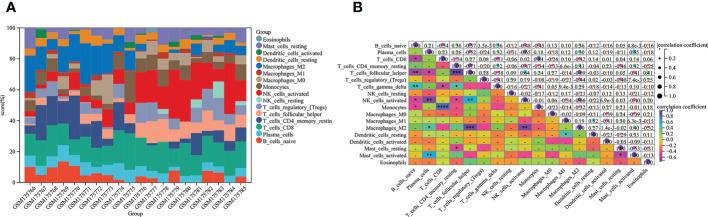
Relationships between immune cells from Cibersort analysis based on GSE7305. **(A)** The abundance values of sixteen immune cells in each sample. **(B)** Eighteen types of immune infiltrates with correlation. The squares in the heat map represent correlation strength, orange represents positive correlation, and blue represents negative correlation. The darker the color, the more meaningful it is. The P value is shown. The darker the color, the smaller the P value, and the lighter the color, the larger the P value (*P < 0.05, **P < 0.01, ***P < 0.001, ****P < 0.0001).

Increasing evidence indicates a dominating immune system contribution to endometriosis pathogenesis (43). Immune cells, particularly T and B lymphocytes and NK cells, appear to be major regular roles in the onset and progression of endometriosis (44, 45). Above results prompted us to further analyze the immune infiltration of 16 kinds of immune cells in endometriosis by Cibersort and the data shown in the histogram (**Figure 2A**). Through correlation analysis, we found that some immune cells interacted with each other. Naive T cells demonstrated multifaceted correlation. By definition, naive B cells were negatively correlated with the estimated abundance of CD8+ T cells, T helper cells, NK cells activated, monocytes, and were positively correlated with γδ-T cells. Antibody-secreting plasma cells were positively associated with M2 macrophages, mast cells activated, and inversely correlated with T cells and NK cells. For CD8+ T cells, monocytes and γδ-T cells were negatively correlated. While for resting CD4+ T cells, T helper cells and NK cells activated were positively, and γδ-T cells were negatively. There were also candidates for reflecting T helper cells were NK cells activated (with positive correlation) and M2 macrophages (with negative correlation). Additionally, M2 macrophages and NK cells activated were negatively correlated, mast cells activated and mast cells resting were negatively correlated (**Figure 2B**). Based on the above results, immune cells and infiltrating immune provide relevant information for endometriosis.

### Immune landscape analysis

**Figure 3:**
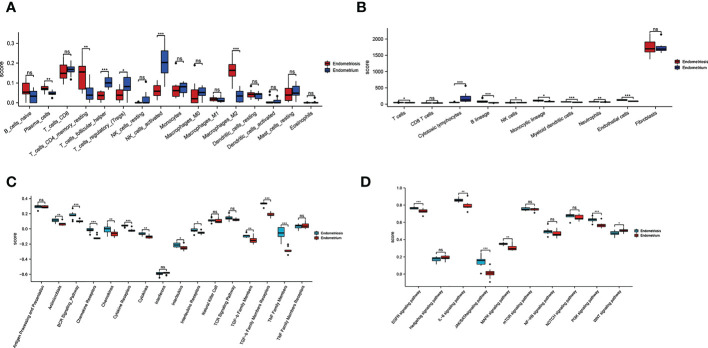
Relative cellular fraction of 16 immune infiltrating cell types in endometriosis assessed by Cibersort **(A)**. Eight immune cell types, endothelial cells, and fibroblasts in endometriosis evaluate by MCP-counter **(B)**. Differential expression of immune response gene **(C)** and immune response signaling pathways **(D)** in endometriosis. *P < 0.05; **P < 0.01; ***P < 0.001; ns, not significant with P > 0.05.

To further investigate differences in immune cell type between endometriosis and non-endometriosis, the types of immune cell composition scores were calculated by Cibersort and MCP-counter. According to Cibersort, there are abundant plasma cells, CD4+ T cells, and M2 macrophages in endometriosis. In contrast, T follicular helper cells, NK cells activated, and T regulatory cells (Tregs) are underexpressed (**Figure 3A**). In comparison, MCP-counter findings demonstrate some commonalities and differences. Uniquely, the absolute abundance score for endothelial cells is higher using MCP-counter (**Figure 3B**). For other eight major immune cell types estimated by MCP-counter, In addition to elevated levels of T cells, other immune cells are broadly expressed, including B cells, monocyte lines, myeloid dendritic cells, and neutrophils. While NK cells score is lower and no differential CD8+ T cells expression between endometriosis and non-endometriosis. Despite the insignificance of CD8+ T cells, cytotoxic lymphocytes were lower in endometriosis compared to normal endometrium. In the immune response gene concentration, antimicrobials, BCR signaling pathway, chemokine receptors, chemokines, cytokines, interleukins, interleukin receptors, TGF-β family members, and TNF family members were highly expressed in endometriosis (**Figure 3C**). In the immune-related signaling pathways, there were several pathways, including “EGFR signaling pathway”, “IL-6 signaling pathway”, “JAK/STAT signaling pathway”, “MAPK signaling pathway”, “PI3K signaling pathway”, enriched in endometriosis. Inactivation of WNT signaling is observed in endometriosis (**Figure 3D**).

### Acquisition and enrichment of DEGs

**Figure 4:**
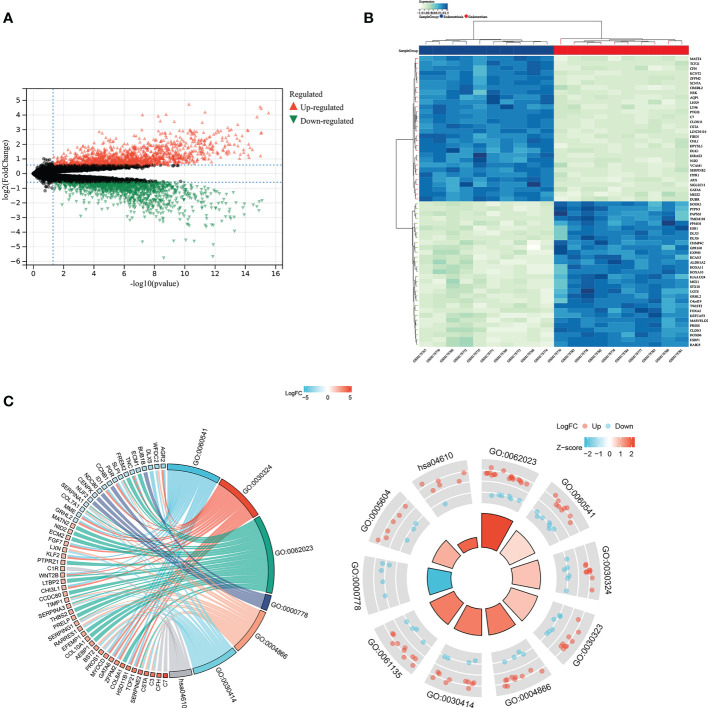
GO and KEGG analysis for identified DEGs. DEGs between endometriosis and non-endometriosis were displayed in volcano plot **(A)** and the top 30 DEGs in the heat map **(B)**. **(C)** The chord diagram and circle diagram showed the enrichment analysis of DEGs.

Through the Sanger assistant platform, we analyzed the GSE7305 dataset. We obtained DEGs, including 144 up-regulated genes and 133 down-regulated genes, displayed by volcano plot (**Figure 4A**) and heatmap (**Figure 4B**) showing the expression of the top 30 DEGs. DEGs were enriched in respiratory system development, lung development, collagen-containing extracellular matrix, condensed nuclear idea kinetochore, endopeptidase inhibitor activity, peptidase inhibitor activity, complement and coagulation cascades. They were significantly enriched through the circle diagram and chord diagram (**Figure 4C**). **Table 1** shows the enrichment analysis results of DEGs in circle and chord plots.

**Table 1:** GO and KEGG enrichment analysis of circle graph and chord graph of DEGs.

Ontology	ID	Description	GeneRatio	BgRatio	p value	p.adjust	q value
BP	GO:0060541	respiratory system development	16/255	198/18670	1.43e-08	2.21e-05	1.89e-05
BP	GO:0030324	lung development	15/255	172/18670	1.47e-08	2.21e-05	1.89e-05
CC	GO:0062023	collagen-containing extracellular matrix	23/260	406/19717	5.04e-09	1.55e-06	1.29e-06
CC	GO:0000778	condensed nuclear chromosome kinetochore	5/260	15/19717	1.03e-06	1.59e-04	1.32e-04
MF	GO:0004866	endopeptidase inhibitor activity	14/247	175/17697	1.71e-07	4.24e-05	3.90e-05
MF	GO:0030414	peptidase inhibitor activity	14/247	182/17697	2.77e-07	4.24e-05	3.90e-05
KEGG	hsa04610	Complement and coagulation cascades	7/124	85/8076	3.11e-04	0.066	0.063

### Identification and enrichment of differentially expressed TFs

**Figure 5:**
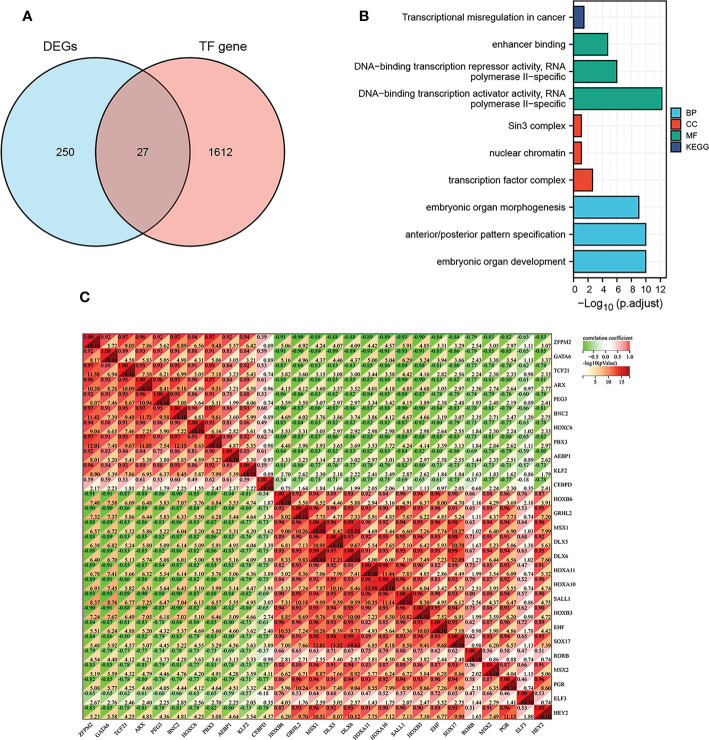
TFs in DEGs and TF-TF interactions. **(A)** Overlap between DEGs and TFs. **(B)** The enrichment analysis of GO and KEGG for twenty-seven TFs regrouped from the intersection. **(C)** The correlation of twenty-seven TFs between each other.

As the crucial role of TFs in endometriosis, we regrouped genes by using the intersection of DEGs and TFs. Twenty-seven TFs were finally taken based on the intersection in the GSE7305 dataset (**Figure 5A**). Twenty-seven genes were submitted for GO and KEGG enrichment analysis. Analysis results appear in **Figure 5B**, including transcriptional misregulation in cancer, enhancer binding, RNA polymerase II-specific, DNA-binding transcription repressor activity, RNA polymerase II-specific, DNA-binding transcription activator activity RNA polymerase II - specific, Sin3 complex, nuclear chromatin, transcription factor complex, embryonic organ morphogenesis, anterior/posterior pattern specification, embryonic organ development. **Figure 5C** shows those twenty-seven target TFs interactions. Except for RORB and CEBPD, ELF3, and CEBPD, the remaining other genes are related to each other.

### Correlation analysis between differentially expressed TFs and immune landscape

**Figure 6:**
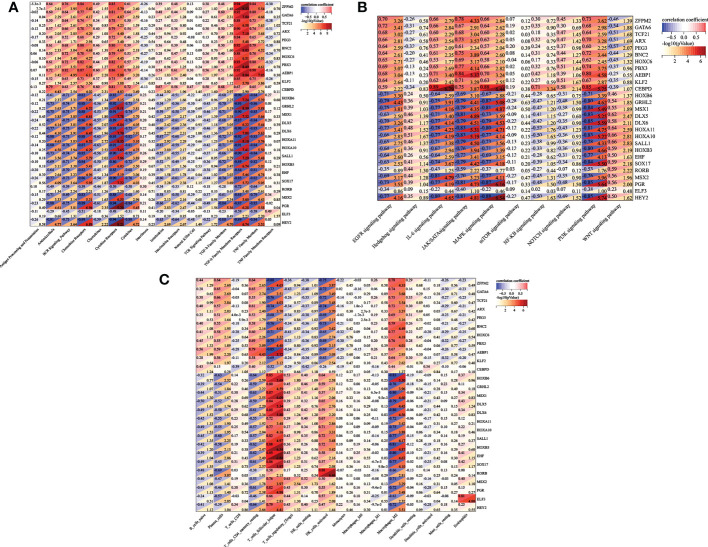
TFs-immune interaction. **(A)** The relationship between the target twenty-seven TFs and immune-related genes, associated immune pathways **(B)**, and immune infiltrating cells **(C)**.

We further wanted to explore the relationship between the target twenty-seven TFs, immune-related genes, and associated pathways. Approximately half of these genes, including GRHL2, HOXB6, MSX1 DLX5, DLX6, HOXA11, HOXA10, SALL1, HOXB3, EHF, SOX17, MSX2, PGR, HEY2, were found negatively correlated with antimicrobial, BCR signaling pathways, chemokine receptors, cytokines, TGF-β family members receptors, and TNF family members (**Figure 6A**, R > 0.6, P < 0.05). As a comparison, some of the TFs, including ZFPM2, GATA6, TCF21, ARX, PEG3, BNC2, HOXC6, PBX3, AEBP1, KLF2, CEBPD, were positively correlated with cytokine receptors, TGF-β family members receptors, and TNF family members (R > 0.6, P < 0.05). In addition, there was a negative relationship between RORB and TGF-β family members and TNF family members (R > 0.6, P < 0.05). There were no significant correlations between ELF3 and immune response genes.

In exploring the relationship between twenty-seven differentially expressed TFs and immune response pathways, most genes were involved in several signaling pathways, such as EGFR signaling pathway, IL-6 signaling pathway, MAPK signaling pathway, and JAK/STAT signaling pathway, and PI3K signaling pathway. The genes including GRHL2 HOXB6, MSX1 DLX5, DLX6, HOXA11, HOXA10, SALL1, HOXB3, EHF, SOX17, MSX2, PGR, HEY2 were negatively correlated (**Figure 6B**, R > 0.6, P < 0.05). While ZFPM2, GATA6, TCF21, ARX, PEG3, BNC2, HOXC6, PBX3, AEBP1, KLF2, and CEBPD showed a positive correlation with EGFR signaling pathway (except CEBPD), IL-6 signaling pathway (except PEG3), MAPK signaling pathway (except HOXC6), JAK/STAT signaling pathway, and PI3K signaling pathway (R > 0.6, P < 0.05).

In terms of immune cell types, among the twenty-seven differentially expressed TFs, we found that ZFPM2, GATA6, TCF21, ARX, PEG3, BNC2, HOXC6, PBX3, AEBP1, KLF2 were negatively correlated with T follicular cell, NK cells activated, and positively correlated with M2 macrophage. Genes including HOXB6, GRHL2, MSX1, DLX5, DLX6, HOXA11, HOXA10, SALL1, HOXB3, EHF, SOX17, MSX2, PGR, ELF3, HEY2 seemed to be positively correlated with T helper cell. The type of NK cell activation showed a positive correlation with HOXB6, DLX5, HOXA11, HOXA10, SALL1, HOXB3 and RORB. HOXB6, GRHL2, MSX1, DLX5, DLX6, HOXA11, HOXA10, SALL1, HOXB3, EHF, SOX17, RORB, MSX2, PGR, ELF3 and HEY2 were negatively correlated with M2 macrophage (**Figure 6C**).

### Screening and diagnostic value of five TFs (AEBP1, DLX6, HOXB6, KLF2 and RORB)

**Figure 7:**
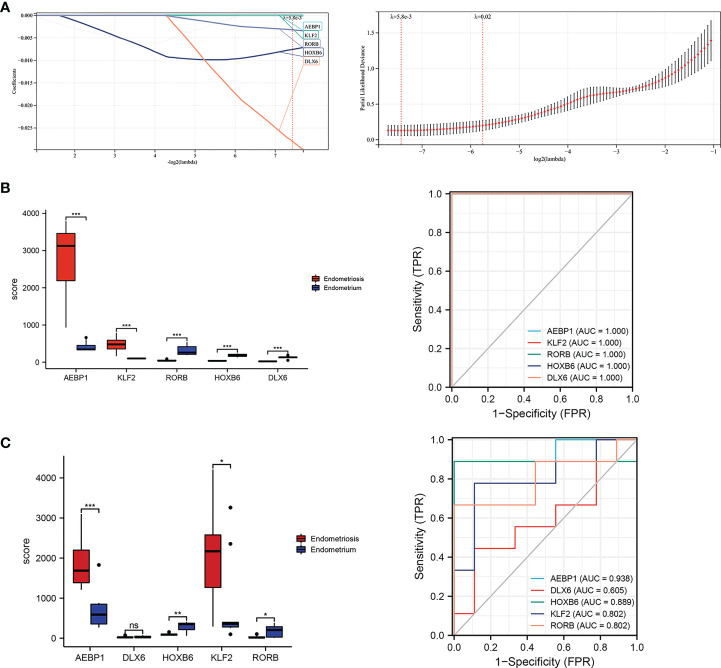
Lassol model screened TFs diagnostic values both in the training and validation cohorts. **(A)** Results of the Lasso multivariate model. **(B)** ROC curves of AEBP1, DLX65, HOXB6, KLF2, and RORB in the GSE7305 dataset. **(C)** ROC curves of AEBP1, DLX65, HOXB6, KLF2, and RORB in the GSE11691 dataset. *P < 0.05; **P < 0.01; ***P < 0.001; ns, not significant with P > 0.05.

**Figure 8:**
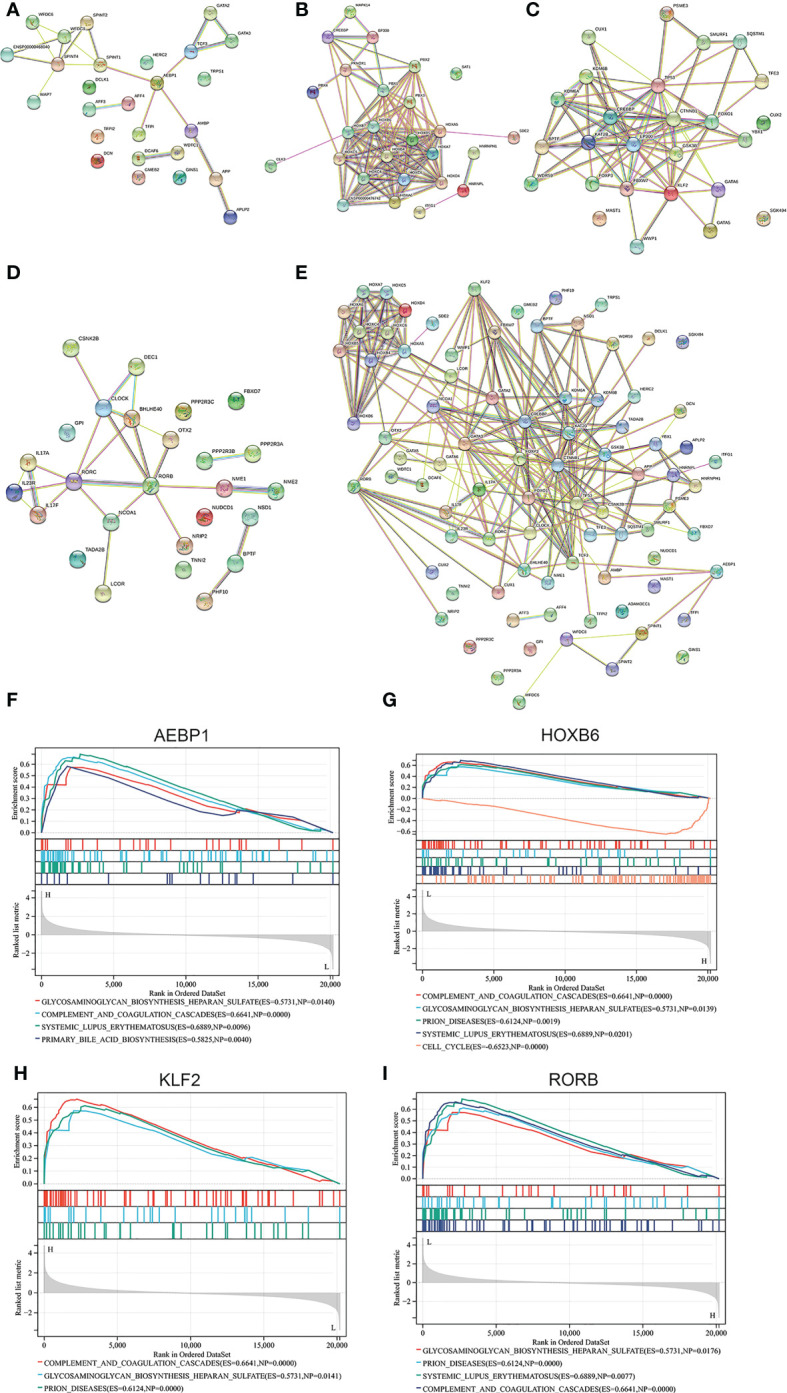
Correlation network generated for AEBP1, HOXB6, KLF2, RORB using String. The interaction network diagram for AEBP1, HOXB6, KLF2 and RORB respectively **(A-D)** and combined comprehensive network diagram **(E)**. **(F-I)** The associated pathway from GSEA analyses for AEBP1, HOXB6, KLF2, and RORB.

Then, we reconstructed twenty-seven TFs expression spectrum. The Lasso method for regression analysis was used to get the optimal model. We set Lambda to 0.00580032815322684 and ended up with five TFs (AEBP1, DLX6, HOXB6, KLF2 and RORB). The risk score of the constructed model was calculated as: (3.87064053496486e-05*AEBP1) + (-2.49026387792545e-05*KLF2) + (-0.00755688231735056*HOXB6) + (-0.0279603209725811*DLX6) + (-0.00321782088846974*RORB) (**Figure 7A**). AEBP1 and KLF2 were higher expressed, while DLX6, HOXB6, and RORB were lower expressed in endometriosis in the GSE7305 dataset. ROC curve analysis for the above five genes revealed that the area under the ROC curve was all 1 (**Figure 7B**). For validation, we referred GSE11691 dataset as validation group. We verified the levels of five genes, and the verification results were similar to those from GSE7305, exclusion DLX6. The area under the ROC curve was separately of AEBP1 = 0.938, DLX6 = 0.605, HOXB6 = 0.889, KLF2 = 0.802, RORB = 0.802 (**Figure 7C**). Target genes had outstanding specificity and sensitivity, excluding DLX6. Therefore, DLX6 was not included for the association analysis. Here, GSE23339 was employed to revalidate the diagnostic value for remaining four TFs. The expression results were consistent with GSE7305 and GSE 11691(**Figure S1A**). The area under the ROC curve was all greater than 0.9 (**Figure S1B**), and correlation network diagrams were generated for AEBP1, HOXB6, KLF2 and RORB using String (**Figures 8A–E**).

### Enriched KEGG pathways of four key TFs in GSEA analysis

Interestingly, in the case of correlation focused on selected four genes, the enriched pathways are similar to that analysis by GSEA for the GSE7305 dataset (**Figure 1C**). Those pathways include glycosaminoglycan biosynthesis heparan sulfate, complement and coagulation cascades, SLE, and primary bile acid (**Figures 8F–I**). Among those, common pathways, including complement cascades, glycosaminoglycan biosynthesis, and heparan sulfate, attracted HOXB6, KLF2, and RORB. SLE, an autoimmune disease, predominantly affecting young females, is implicated in connection with endometriosis (46). Our results showed that HOXB1, AEBP1, and RORB were involved in endometriosis and SLE.

### The Co-expression modules for four key TFs clusters analyzed by WGCNA

**Figure 9:**
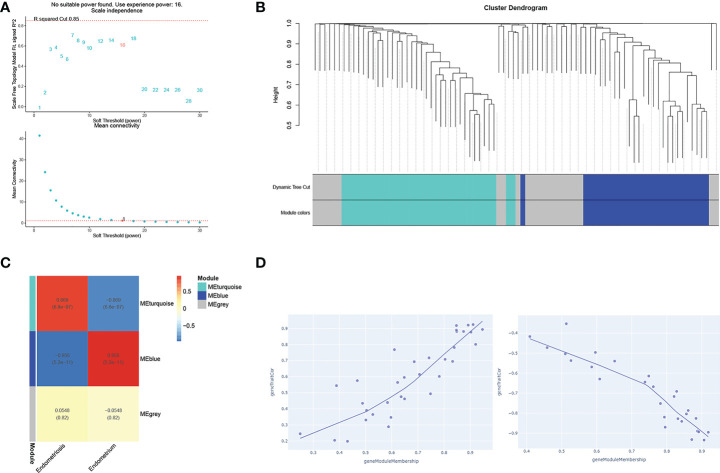
WGCNA analysis of gene networks for closely related four genes (AEBP1, HOXB6, RORB, and KLF2). **(A)** The soft thresholds. **(B)** A tree of modules, each color represents a specific coexpression module and the upper branches represent genes. Genes that do not belong to any module will be marked grey. **(C)** The heat maps of different co-expression modules. **(D)** The turquoise module (uptrend) and the blue module (downtrend), respectively.

The PPI network was constructed with four selected TFs based on the String database. We obtained eighty-five co-expressed gene pairs (**Figure 8E**). Closely related four-gene clusters make us investigate the co-expression modules in endometriosis using WGCNA. The introduction of a soft threshold in network topology affects the network’s scale independence and mean connectivity. A soft threshold of 16 in the GSE7305 dataset was used to obtain the approximate scale-free topology, and the scale-free topology fit index >0.85 was considered as the lowest power (**Figure 9A**). Module-trait correlation analyses showed that two modules were related to endometriosis (**Figure 9B**). The results of turquoise module (r=0.869, P=6.0e-07) and blue module (r=-0.956, P=5.2e-11) were significantly correlated with the incidence of endometriosis (**Figure 9C**). The scatter plot shows the linear relationship of genes in the meaningful correlation module of endometriosis (**Figure 9D**).

### GO and KEGG in metascape

**Figure 10:**
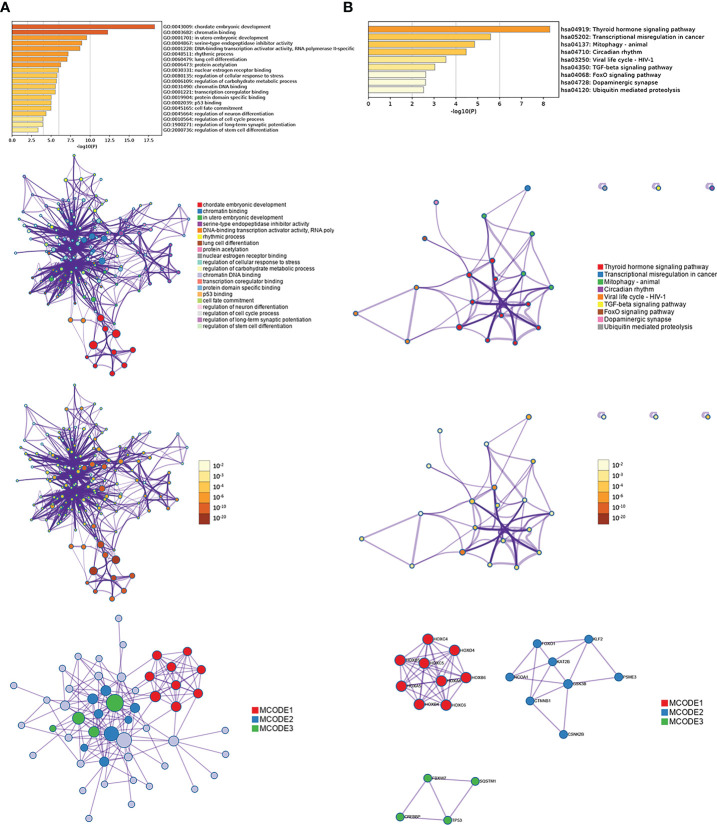
Enrichment analysis of interaction network by Metascape for sixty-two genes extracted from WGCNA, including GO **(A)** and KEGG **(B)**.

Sixty-two genes were extracted from eighty-five genes after WGCNA analysis. To understand the biological functions of the sixty-two co-expression genes, enrichment analyses based on GO and KEGG were inspected using Metascape. GO-term analysis revealed that those sixty-two extracted genes were implicated in many aspects, including chordate embryonic development, chromatin binding, *in utero* embryonic development, serine-type endopeptidase inhibitor activity, DNA-binding transcription activator activity, RNA polymerase II-specific rhythmic process, lung cell differentiation, and protein acetylation (**Figure 10A**). KEGG pathway analysis of the sixty-two genes showed pathways involved in thyroid hormone signaling pathway, transcriptional misregulation in cancer, mitophagy-animal, circadian rhythm, viral life cycle-HIV-1, and TGF-β signaling pathway (**Figure 10B**). In addition, three modules were established through Metascape, and KLF2 and HOXB6 genes were again present in two modules (**Figure 10B**), implying important biomarkers in endometriosis.

### SVM-RFE and random forest models validation

We further applied other machine learning approaches to validate whether HOXB6 and KLF2 would be repeated occurrence in various modules, such as SVM-RFE, random forest algorithm. 15 genes were confirmed from 27 TFs by SVM-RFE model, including HOXB6, ELF3, RORB, HOXC6, BNC2, ZFPM2, TCF21, PEG3, ARX, GATA6, HOXB3, KLF2, EHF, MSX1, SALL1 (**Figure S2A**). **Figure S2B** displayed 15 genes with the largest contribution to the random forests model among 27 TFs, they were HOXC6, GATA6, AFPM2, KLF2, HOXB6, ARX, PBX3, AEBP1, HOXB3, MSX1, TCF21, BNC2, HOXA10, HOXA11, DLX5. Lastly, the HOXB6 and KLF2 were the concordant TFs according to the Lasso, SVM-RFE, random forest algorithms (**Figure S2C**). Thus, HOXB6 and KLF2 were stable and valuable biomarkers across various models for endometriosis.

### Prediction lncRNAs-miRNAs-mRNAs network of TFs regulation

**Figure 11:**
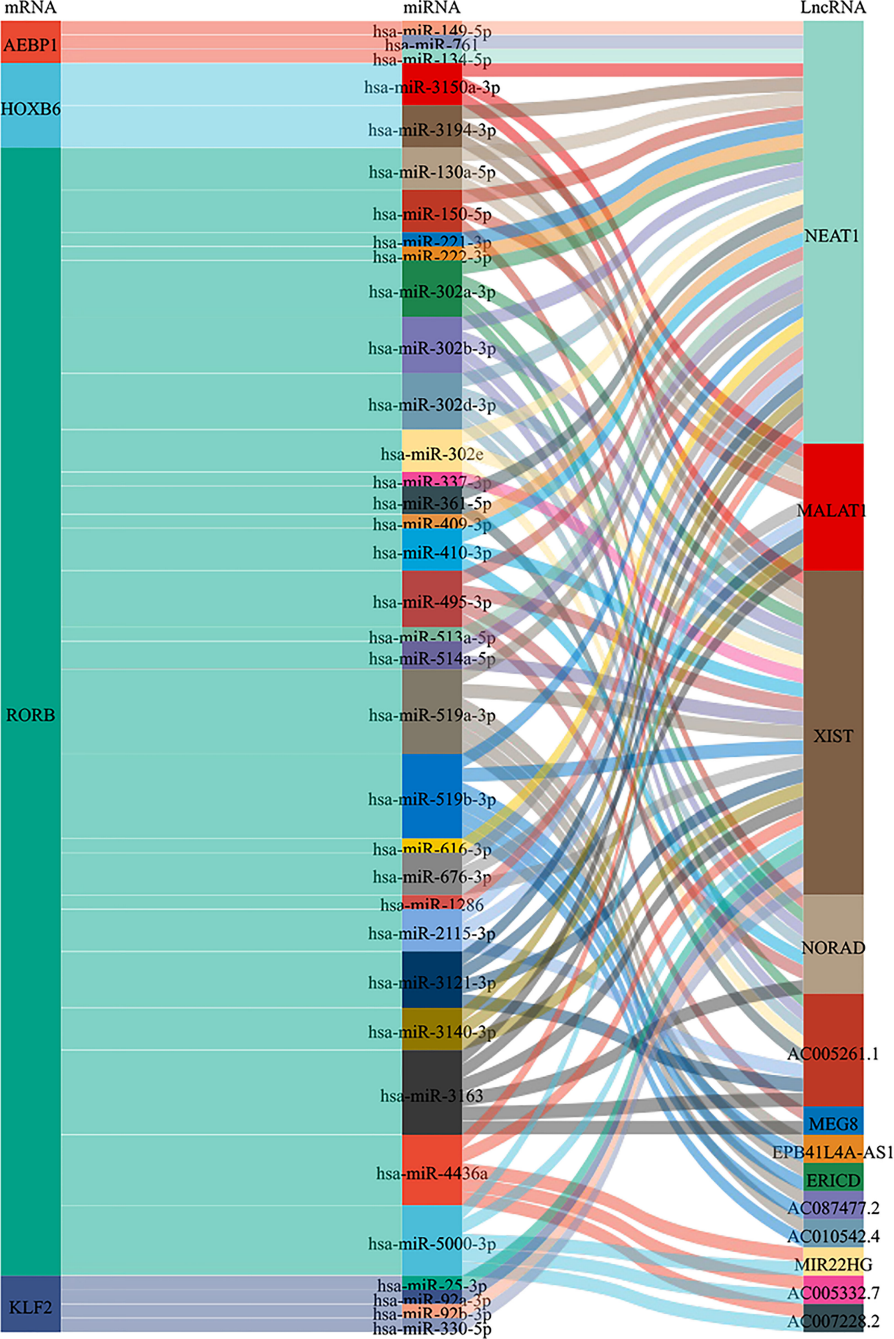
Sankey diagram of the lncRNAs-miRNAs-mRNAs network. Each rectangle represents a gene, and the size of the rectangle indicates the degree of connectivity of each gene.

To gain insight into the regulation of the AEBP1, HOXB6, RORB, and KLF2, we examined upstream regulation and screened microRNAs and lncRNAs for targeting these four genes. miRNAs predicted by Diana-MicroT, miRWalk, Starbase, TargetScan, and miRDB database. Extracted 3, 2, 26 and 4 upstream miRNAs were found to target AEBP1, HOXB6, RORB and KLF2, respectively. In addition, lncRNA-miRNA relationships for four gene-related miRNAs were predicted by StarBase, among which lncRNAs were 1, 3, 13 and 1, respectively (**Figure 11**).

### Favorable affinity of four key TFs for goserelin and dienogest

**Figure 12:**
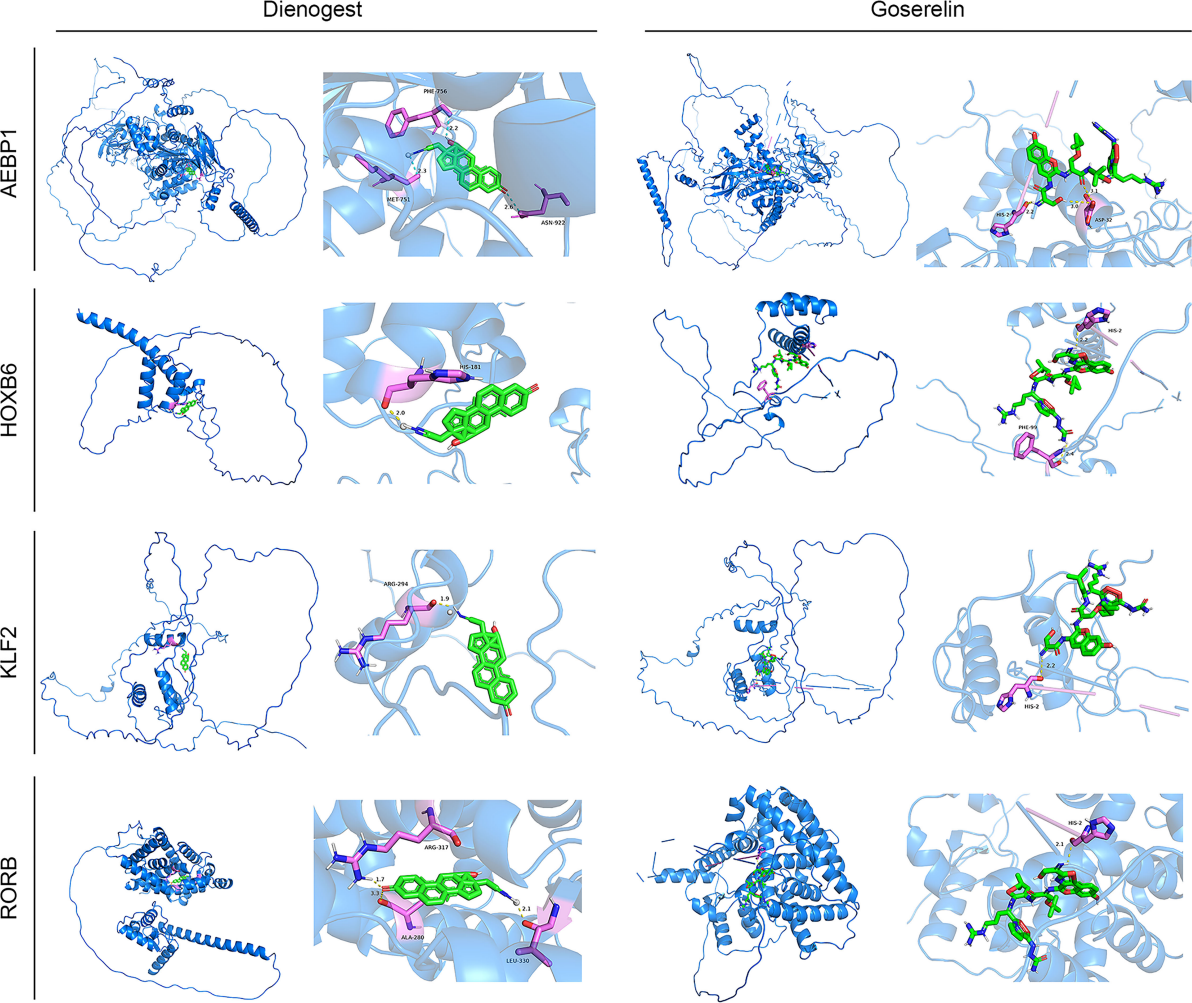
Results of molecular docking simulations. Protein-ligand docking pocket for each transcription factor (AEBP1, HOXB6, KLF2 and RORB). The pocket with the lowest binding energy when the transcription factor binds Dienogest or Goserelin. PHE, Phenylalanine residue; MET, methionine; HIS, Histidine residue; ARG, Arginine; ALA, alpha Linolenic acid; LEU, leucine; ASN, ASP, Aspartic acid.

Four screen TFs have shown an excellent diagnostic value in endometriosis. Finally, it is ideal to identify whether those four TFs harbor meaningful therapeutic value. Goserelin and dienogest are considered as first-line drug therapy for endometriosis (47). Based on the structure of four proteins, molecular docking was performed to predict the binding site of four target genes on goserelin and dienogest. The lowest binding energy of the candidate drugs with four proteins was all less than -1.2 kcal mol-1, indicating they had a good affinity (**Table 2**). Then, small molecule drug docking targets with the lowest binding energy were selected for docking visualization (**Figure 12**). The docking results showed that both goserelin and dienogest could bind to the active pockets of four core target proteins and form hydrogen bond interactions with surrounding amino acid residues.

**Table 2:** The lowest binding energy (kcal/mol) for molecular docking.

Drug	Target			
	AEBP1	HOXB6	KLF2	RORB
Goserelin	-10.78	-2.68	-1.41	-3.67
Dienogest	-7.77	-6.38	-6.97	-9.69

## Discussion

In most cases, endometriosis is one of the leading causes of pelvic pain and infertility. It is a global health problem for women. The discovery of immune dysfunction and abundance of estrogen theories are crucial for understanding the growth of endometriosis. However, we still have limited insight into it. Therefore, in-depth study could be helpful for diagnosis, treatment, progression, and re-treatment for endometriosis.

GSVA quantified gene enrichment results by calculating the enrichment fraction of specific gene sets in each sample. GSEA divided the samples in the input gene expression matrix into two groups and sorted all genes. Firstly, we conducted enrichment analysis of the GSE7305 dataset by both methods (**Figure 1B, C**), combination GSEA analysis of four core TFs obtained from multiple screening (**Figure 8F, G, H, I**). The results bear a substantial similarity: pathways enriched in glycosaminoglycan biosynthesis heparan sulfate, complement and coagulation cascades, and SLE, maybe focus on immunity and metabolism. The analysis results have drawn our attention to SLE, which appears multiple times. As is known to all, SLE is an autoimmune disorder that can affect multiple organs. Most patients could be detected antinuclear antibodies (ANA), and studies have shown that 18% of endometriotic patients with ANA, which may indicate that endometriosis and SLE have certain relevance (48–50). Recent studies also indicated that patients with endometriosis suffer a higher prevalence of autoimmune disease (51–53). Previous works mainly focus on retrospectives and meta-analyses. However, they have not provided strong evidence to support the notion that increased risk of prevalence for each other. Our analyses point to, HOXB6, KLF2, and RORB, maybe cross-regulatory genes in endometriosis and SLE.

Immunological dysfunction was considered a critical promoter of endometriosis. However, whether immune dysfunction is a cause or a consequence of endometriosis could not be clarified, and which immune infiltrating cells are dominant in endometriosis is not yet clear. Cibersort and MCP-counter were both utilized to estimate immune cell subtypes for GSE7305. What are obvious common results between the two methods are that NK cells activated were suppressed, B cells activated and M2 macrophage cells were accumulated, and no differential CD8+ T cells expression in endometriosis (**Figure 3A, B**). There is consensus that NK cell activity has been suppressed in pelvic endometriosis (54, 55), which leads to immune escape of endometriotic fragments. For specialized GSE7305 database of ovarian endometrioma, NK cell results are consistent with previous endometriosis analyses. Endometriosis is often defined as an autoimmune disorder and anti-endometrial antibody has been verified in the serum and pelvic fluid (56). Endometriotic patients with autoantibodies develop coexistence of various autoimmune diseases such as inflammatory bowel disease and allergies (57, 58). So, studies indicated that antibody-secreting plasma cells increased in endometriosis (59). Plasma cells were obviously increased in ovarian endometrioma (P<0.01), and B native cells were no different in our studies.

Similarly, pooled data from twenty-two studies pointed no difference for B cells (59). Maybe for B cells, type B cells are fundamentally different in endometriosis, suggesting further investigation should refine the classification of B cells. Activated macrophages are well classified as M1 and M2 macrophages. Studies tended to think the equilibrium between M1 and M2 macrophages was lost, and alternatively activated M2 macrophage was dominant, mainly in pelvic fluid and peritoneal endometriosis (60, 61). In our data, there are abundant M2 macrophage cells in ovarian endometrioma, which implies not only peritoneal endometriosis, but also the subtype of ovary harbored increased M2 macrophage. Regarding DCs, the role of DCs in endometriosis function remains controversial. DCs changes, such as the number decreased (62), increased (63) and no changes (64), have been observed in endometriosis patients’ peritoneal fluid. In our analysis, myeloid DCs were increased dramatically by MCP-counter. By using MCP-counter, as expected, endothelial cells (ECs) numbers are significantly increased in ovarian endometrioma. Endothelial cells are essential regulators for angiogenesis, which is deeply involved in regulating endometriosis.

Immune-related gene expression signatures for GSE7305 in our results were the diversity, mainly in BCR signaling pathway, chemokine receptors, chemokines, cytokines, cytokine receptor, TGF-β family members, TGF-β family members receptor, and TNF family members (**Figure 3C**, P<0.01, P<0.001). Most of these can be classified as cytokines, central mediators in the immune pathway and response. Măluţan et al. reported that serum levels of IL-1, IL-4 and IL-10 in endometriosis were significantly higher than in the control group (65). Endometriosis patients also display increased IL-1β, IL-6, and TNFα (66, 67). The TGF-β superfamily makes up over thirty ligands in humans. In recent years, a growing consensus has been reached that increased levels of TGF-β are present in ectopic lesions, serum, and peritoneal fluid of endometriosis patients. Increased TGF-β was involved in ectopic cell survival, angiogenesis, attachment, and invasion, together with decreased immune cell activity (68–71). Here, our results are consistent with the previous study. However, future studies are needed to further elucidate how cytokines contribute to changes in endometriosis.

In the discussion of immune response signaling pathways, we found that EGFR signal pathway, IL-6 signal pathway, JAK/STAT signal pathway, MAPK signal pathway, and PI3K signal pathway were highly expressed. In contrast, the WNT signal pathway was low expressed in endometriosis (**Figure 3D**). As a multifunctional cytokine, IL-6 has been found to increase its concentration in ectopic endometrial tissue both in peripheral blood and peritoneal fluid (72) and promote endometrial cell proliferation (73). IL-6 levels decrease after surgery or GnRH agonists are used to treat endometriosis (74). MAPK/ERK and PI3K/AKT signaling pathways are intracellular kinases that activate the endometriotic environment and exert anti-apoptotic functions (75). JAK/STAT pathway is responsible for cytokine transduction and is involved in cell proliferation, migration, differentiation, and immune regulation (76, 77). Constitutively activated kinase signals are found in endometriosis and investigated as therapeutic targets. Sulforaphane attenuates endometriosis inflammation by inhibiting PI3K/AKT signaling Pathway (78). Tofacitinib effectively reduced STAT3 phosphorylation in Ishikawa cells and in human primary stromal and epithelial cells in patients with or without endometriosis (79). Abnormal endometrial WNT/β-catenin signaling can lead to endometriosis and endometrial cancer. In the mouse uterus, a range of down-regulated WNT/β-catenin target genes impelled embryo implantation, which could be reversed by blocking β-catenin degradation (80). To address the therapy challenges, the pathway level may provide complementary information for exploring potential therapeutic targets for endometriosis.

Past researches have shown that TFs, such as nuclear receptors superfamily and pro-inflammatory transcription factor family, play a pivotal role in driving endometriosis (81–84). For nuclear receptors of ligand-activated TFs, excessive levels of human estrogen receptor beta (ESR2) and deficiency in progesterone receptor (PGR) was central to understanding pathogenesis. The overexpression of hypomethylated GATA binding protein 6 (GATA6) made normal endometrial stromal cells develop endometriotic phenotypes and inhibited hormone sensitivity. Forkhead Box O1 (FOXO1) level in endometriosis was 1.6 times lower in the early secretory phase (15-21 days) than in the normal group. While during the proliferative phase of the menstrual cycle, another transcription factor of c-Jun mRNA level was higher in endometriosis (1.5 times) (85). Transcription factor 21 (TCF21), a member of the basic helix-loophelix (bHLH) TF, was required for triggering endometriotic fibrosis (86). Therefore, exploring the role of TFs in endometriosis is extremely important. TFs were obtained from the human transcription factor database, and twenty-seven differentially expressed TFs were obtained by intersection with DEGs in GSE7305. Twenty-seven TFs are extremely closely related, most of which are related to immune-related genes, associated immune pathways, and immune infiltrating cells (**Figure 6**).

By the Lasso model, we further screened five genes (KLF2 AEBP1, DLX6, HOXB6, RORB). A prognostic gene prediction model was proposed and verified both in GSE7305 and GSE11691database. Five genes, except DLX6, the ROC values of AEBP1, HOXB6, KLF2 and RORB were all greater than 0.8 (**Figure 7B, C**), suggesting the high diagnostic value of the four genes. Then, four genes created a connected module in the PPI network, and they were closely connected in the PPI network (**Figure 8E**). We then employed WGCNA to redefine specific gene modules, and sixty-two genes were lastly reselected. Enrichment analyses (Go and KEGG) of sixty-two genes were performed using Metascape. Three new sets of modules were identified, and KLF2 and HOXB6 genes were again present in two of them. Besides, SVM-RFE and random forest algorithms identified that KLF2 and HOXB6 were the coincident genes, suggesting dominant contribution to endometriosis.

To our knowledge, there were no targeted researches for four screened TFs in endometriosis. A study focused on GSE51981, containing four stages of endometriosis and normal endometrium, pointed that the mRNA levels of AEBP1 and HOXB6 shifted in I/II, III/IV stages. AEBP1 and HOXB6, together with IGF-1, CYP11A1, MMP-2, CC2D2A, IER3, STX18, maybe staging markers for endometriosis (87). Moreover, in comprehensive analyses for GSE120103 and GSE105764 datasets, Li, Q et al. found that HOXB6 was abnormal expressed not only in endometriosis but also in infertile group (88). The roles of KLF2 and RORB in endometriosis have not been explored. Our finding highlight the immune correlates for four TFs. Among the immune response pathways, prediction of Phosphatidylinositol 3-kinase (PI3K), The Janus kinase (JAK)/signal transducer of activation (STAT), mitogen-activated protein kinase (MAPK) and interleukin-6 (IL-6) signal pathways are the central pathways of four TFs (**Figure 6B**). PI3K pathway is commonly activated by cytokines, antigen, and molecules. PI3K, through IL-1R and TNFR, participates innate immunity and inflammation (89). Eutopic and ectopic endometrial cells had high expression of pAKT and increased level of AKT caused PR reduced in endometriosis (90, 91). JAK/STAT controlled more than 50 cytokines and growth factors, it serves as central position for immune regulation. Mutation of JAK/STAT components are causative for various immunological phenotypes in human (92–94). Eutopic endometrium with upregulated phosphorylated STAT3, and overactivity of STAT3 impacted ectopic lesions survival (95). The MAPK signaling consists of a group of serine/threonine kinase pathways, at least three tiers: MAP3K, MAP2K, and MAPK. Phosphorylation and dephosphorylation are the main regulated manner. MAPK family members have been reported to respond to inflammatory cytokines and immune regulation (96, 97). In endometriotic cells, ERK is capable of activating by a range of cytokines, such as TNFa, IL-1β, TGFβ, MCP1 (98–100). TNFα employs ERK and enhance the IL-6 and IL-8 levels (98) and IL-17 also causes IL-8 secretion through p38 MAPK pathways in ectopic cells (101). There are some previous reports of four TFs involved in above immune related signals in other diseases, their possible roles for immunophenotype in endometriosis have not been reported. From perspective of immune cell types, M2 type macrophages and NK cells activated reduction are the dominant types of the immune response in endometriosis (55, 60). In our results, M2 macrophage cells is positively correlated with AEBP1, KLF2 and negatively correlated with HOXB6 (R<-0.8), RORB (**Figure 6C**). NK cells activated correlates positively with HOXB6 and RORB (R>0.8) and negatively with KLF2, AEBP1. HOXB members extensively regulate immune functions in cancer (102, 103). CXCL8 secreted from tumor associated macrophage *via* HOXB13 promoted endometrial cancer cells invasion (104). HOXB3 intervened early lymphoid and myeloid development (105). Appropriate cytokines stimulated, HOXB8 together with estrogen receptor were able to construct immortalized macrophages or neutrophils models (106). Unlike the other HOXB members, the HOXB6 for immunophenotypes were not well defined, especially for macrophage. Given reported researches on the HOXB family members of the immune regulation, it should be possible to explore the HOXB6 in endometriosis. In the course of study for immune related genes, the most notable relationships for four TFs are the TGF-β family member receptors and TNF family members (**Figure 6A**), especially for AEBP1 and HOXB6 (R>0.8). AEBP1 was considered as a new proinflammatory mediator. Its overexpression in macrophage, through hedgehog and NF-κB pathways, maintaining an inflammation-cancer microenvironment (107). In addition, AEBP1 was predicted to a downstream targets of TGF-β1 signaling (108). Previous studies rarely provided exact relation for HOXB6 and TGF-β family member receptors, TNF family members. Still in bioinformatics prediction, the studies of four TFs on immune regulation for endometriosis deserve further in-depth investigation.

Based on the ceRNA regulatory network theory, we constructed the lncRNAs-miRNAs-mRNAs network. AEBP1, HOXB6, RORB and KLF2 corresponded to upstream 3, 2, 26 and 4 miRNAs. In addition, lncRNA-miRNA relationships for four genes correspond to 1, 3, 13 and 1 lncRNA, respectively. The lncRNA-miRNA-mRNA network helps us to understand the regulatory mechanism of diseases better. In our results, NEAT1and XIST has the highest binding. NEAT1 may regulate the expressions of AEBP1, HOXB6 and RORB, while XIST may regulate the expressions of HOXB6, RORB and KLF2. Studies have shown that NEAT1 is significantly up-regulated in endometriosis and promotes malignant behavior in endometriosis by targeting miR-124-3p expression (109). XIST expression is low in endometrial tissues and downregulates the expression of PI3K/AKT (110). NEAT1 and XIST may regulate the expression of four TFs (AEBP1, HOXB6, RORB, and KLF2) through the lncRNA-miRNA-mRNA network and participate in the development of endometriosis. Although some studies have been carried out on the regulation mechanism of NEAT1 and XIST in endometriosis, it still needs to be fully explored in the future, especially in the lncRNA-miRNA-mRNA network.

The analytical approach of molecular docking to predict potential drugs has been widely used in bioinformatics, which uses limited resources to find potentially powerful drugs. Goserelin and dienogest are the first choices for treating endometriosis in non-surgical therapy (47). Based on the binding energy of the AEBP1, HOXB6, KLF2 and RORB, they all show good affinity with goserelin and dienogest, suggesting potential therapeutic targets for goserelin and dienogest. However, the specific regulatory mechanism of AEBP1, HOXB6, KLF2, and RORB by goserelin and dienogest remains to be further studied.

## Conclusions

In summary, we explored the role of the immune system and specific signaling pathways in the development of endometriosis from the GSE7305 database. We focused on TFs and screened twenty-seven differential expression of TFs, which were widely involved in immune cell infiltration, immune response, and interacted closely with each other. After the diagnostic validation of five TFs screened from the Lasso model (ROC > 0.8), we identified four core genes (AEBP1, HOXB6, RORB, and KLF2). Then, two of four TFs (KLF2, HOXB6) repeat occurrence in the following multiple modules (String, WGCNA), implying dominant contribution to endometriosis. In the construction of a ceRNA network composed of four target genes, we found that NEAT1 and XIST have the highest degree of binding to miRNA and may regulate the expression of four genes in endometriosis. Finally, in molecular docking, we found that goserelin and dienogest may be potential drugs to control AEBP1, HOXB6, KLF2 and RORB. Above results suggest that four genes may be responsible for potential diagnostic biomarkers and drug targets, while HOXB6 and KLF2 may have higher potential significance.

## Data availability statement

The datasets presented in this study can be found in online repositories. The names of the repository/repositories and accession number(s) can be found in the article/**Supplementary Material**.

## Author contributions

RG conceived the concept, guided the research, and wrote the manuscript. XH performed data analysis. LL, XG, and QW validated the results. YZ revised the manuscript. XLG supervised the whole research. All authors contributed to the article and approved the submitted version.

## Funding

This research was supported by the National Natural Science Foundation of China (81901453).

## Acknowledgments

We thank the authors of the GSE7305, GSE11691 and GSE23339 datasets for their selfless contribution; we thank the free online platform of Sangerbox tools; we thank Dr. Shougang Liu for providing the immune response gene sets and immune response pathways data.

## Conflict of interest

The authors declare that the research was conducted in the absence of any commercial or financial relationships that could be construed as a potential conflict of interest.

## Publisher’s note

All claims expressed in this article are solely those of the authors and do not necessarily represent those of their affiliated organizations, or those of the publisher, the editors and the reviewers. Any product that may be evaluated in this article, or claim that may be made by its manufacturer, is not guaranteed or endorsed by the publisher.
