# Supplementary material for: Gene expression analysis in endometriosis: Immunopathology insights, transcription factors and therapeutic targets
Source: Front Immunol. 2022 Nov 30;13:1037504. doi: 10.3389/fimmu.2022.1037504 (PMC9748153; doi:10.3389/fimmu.2022.1037504)
Supplement: Supplementary file 1 [file DataSheet_1.zip › Raw data and code/Figure12/docking_result.docx]

Experimental structures of four proteins: None available in the PDB.

Downloaded form: [AlphaFold Protein Structure Database (ebi.ac.uk)](https://alphafold.ebi.ac.uk/) (AlphaFold DB provides open access to 992,316 protein structure predictions for the human proteome and other key proteins of interest, to accelerate scientific research.)

- Protein: Adipocyte enhancer-binding protein 1, Gene: AEBP1, UniProt: Q8IUX7
- Protein: Homeobox protein Hox-B6, Gene: HOXB6, UniProt: P17509
- Protein: Krueppel-like factor 2, Gene: KLF2, UniProt: Q9Y5W3
- Protein：Nuclear receptor ROR-beta，Gene：RORB，UniProt：Q92753

2D Structure of Small molecure Downloaded form: https://pubchem.ncbi.nlm.nih.gov/

- Goserelin, PubChem CID: 5311128
- Dienogest, PubChem CID: 68861

**Table. the lowest binding energy (kcal/mol) for molecular docking.**

| Drug | Target | | | |
| --- | --- | --- | --- | --- |
|  | AEBP1 | HOXB6 | KLF2 | RORB |
| Goserelin | -10.78 | -2.68 | -1.41 | -3.67 |
| Dienogest | -7.77 | -6.38 | -6.97 | -9.69 |
