# Supplementary material for: Gene expression analysis in endometriosis: Immunopathology insights, transcription factors and therapeutic targets
Source: Front Immunol. 2022 Nov 30;13:1037504. doi: 10.3389/fimmu.2022.1037504 (PMC9748153; doi:10.3389/fimmu.2022.1037504)
Supplement: Supplementary file 1 [file DataSheet_1.zip › Raw data and code/Figure4/GO and KEGG of chordal graph.docx]

| Ontology | ID | Description | GeneRatio | BgRatio | pvalue | p.adjust | qvalue |
| --- | --- | --- | --- | --- | --- | --- | --- |
| BP | GO:0060541 | respiratory system development | 16/255 | 198/18670 | 1.43e-08 | 2.21e-05 | 1.89e-05 |
| BP | GO:0030324 | lung development | 15/255 | 172/18670 | 1.47e-08 | 2.21e-05 | 1.89e-05 |
| CC | GO:0062023 | collagen-containing extracellular matrix | 23/260 | 406/19717 | 5.04e-09 | 1.55e-06 | 1.29e-06 |
| CC | GO:0000778 | condensed nuclear chromosome kinetochore | 5/260 | 15/19717 | 1.03e-06 | 1.59e-04 | 1.32e-04 |
| MF | GO:0004866 | endopeptidase inhibitor activity | 14/247 | 175/17697 | 1.71e-07 | 4.24e-05 | 3.90e-05 |
| MF | GO:0030414 | peptidase inhibitor activity | 14/247 | 182/17697 | 2.77e-07 | 4.24e-05 | 3.90e-05 |
| KEGG | hsa04610 | Complement and coagulation cascades | 7/124 | 85/8076 | 3.11e-04 | 0.066 | 0.063 |
