# Supplementary material for: Gene expression analysis in endometriosis: Immunopathology insights, transcription factors and therapeutic targets
Source: Front Immunol. 2022 Nov 30;13:1037504. doi: 10.3389/fimmu.2022.1037504 (PMC9748153; doi:10.3389/fimmu.2022.1037504)
Supplement: Supplementary file 1 [file DataSheet_1.zip › Raw data and code/Figure4/The code of chordal graph.docx]

library(clusterProfiler)

library(org.Hs.eg.db) ## org.Mm.eg.db

library(GOplot)

data <- read.table("~/file.txt", header = T)

head(data)

# id logFC

# 1 NAT1 2.921874

# 2 ADH1B 2.104560

# 3 BIRC5 2.879421

# 4 AQP9 2.918583

# 5 BCL2A1 2.116960

# 6 BMP4 2.334144

gene_ids = bitr(geneID = data$id, fromType = "SYMBOL",

toType = "ENTREZID", OrgDb = "org.Hs.eg.db")

head(gene_ids)

# SYMBOL ENTREZID

# 1 NAT1 9

# 2 ADH1B 125

# 3 BIRC5 332

# 4 AQP9 366

# 5 BCL2A1 597

# 6 BMP4 652

ego <- enrichGO(gene = gene_ids$ENTREZID, OrgDb = "org.Hs.eg.db",

keyType = "ENTREZID", ont = "ALL", readable = T)

ego

# #

# # over-representation test

# #

# #...@organism Homo sapiens

# #...@ontology GOALL

# #...@keytype ENTREZID

# #...@gene chr [1:209] "9" "125" "332" "366" "597" "652" "730" "771" "776" "820" "890" "891" "983" "991" "1062" "1101" "1111" "1307" "1308" "1311" ...

# #...pvalues adjusted by 'BH' with cutoff <0.05

# #...232 enriched terms found

# 'data.frame': 232 obs. of 10 variables:

# $ ONTOLOGY : chr "BP" "BP" "BP" "BP" ...

# $ ID : chr "GO:0140014" "GO:0000280" "GO:0048285" "GO:0000070" ...

# $ Description: chr "mitotic nuclear division" "nuclear division" "organelle fission" "mitotic sister chromatid segregation" ...

# $ GeneRatio : chr "32/196" "34/196" "35/196" "23/196" ...

# $ BgRatio : chr "264/18670" "407/18670" "449/18670" "151/18670" ...

# $ pvalue : num 7.53e-25 4.63e-21 1.10e-20 2.23e-20 2.61e-19 ...

# $ p.adjust : num 2.33e-21 7.16e-18 1.14e-17 1.72e-17 1.61e-16 ...

# $ qvalue : num 2.01e-21 6.16e-18 9.78e-18 1.48e-17 1.39e-16 ...

# $ geneID : chr "BIRC5/BMP4/CCNB1/CDC20/CENPE/CHEK1/KIF11/KIFC1/MAD2L1/MYBL2/NEK2/AURKA/TTK/PRC1/AURKB/PTTG1/TRIP13/KIF23/DLGAP5"| __truncated__ "BIRC5/BMP4/CCNB1/CDC20/CENPE/CHEK1/KIF11/KIFC1/MAD2L1/MYBL2/NEK2/AURKA/TOP2A/TTK/PRC1/AURKB/PTTG1/TRIP13/KIF23/"| __truncated__ "BIRC5/BMP4/CCNB1/CDC20/CENPE/CHEK1/KIF11/KIFC1/MAD2L1/MAPT/MYBL2/NEK2/AURKA/TOP2A/TTK/PRC1/AURKB/PTTG1/TRIP13/K"| __truncated__ "CCNB1/CDC20/CENPE/KIFC1/MAD2L1/NEK2/TTK/PRC1/AURKB/PTTG1/TRIP13/KIF23/DLGAP5/NDC80/TACC3/NCAPH/KIF4A/NUSAP1/CDC"| __truncated__ ...

# $ Count : int 32 34 35 23 24 28 20 24 17 19 ...

# #...Citation

# Guangchuang Yu, Li-Gen Wang, Yanyan Han and Qing-Yu He.

# clusterProfiler: an R package for comparing biological themes among

# gene clusters. OMICS: A Journal of Integrative Biology

# 2012, 16(5):284-287

#### enrichKEGG(gene = gene_ids$ENTREZID, organism = "hsa", keyType = "ENTREZID")

### GOplot

ego2 <- as.data.frame(ego)

ego2 <- ego2[,c("ONTOLOGY", "ID", "Description", "geneID", "p.adjust")]

ego2$geneID <- gsub("/", ", ", ego2$geneID)

colnames(ego2) <- c("Category", "ID", "Term", "Genes", "adj_pval")

colnames(data) <- c("ID", "logFC")

circ <- circle_dat(ego2, data)

## Bar Plot

GOBar(subset(circ, category == 'MF'))

## Bubble Plot

GOBubble(circ, title = 'Bubble plot', colour = c('orange', 'darkred', 'gold'), display = 'multiple', labels = 3)

## Circle Plot

GOCircle(circ, table.legend = F)

## Chord Plot

chord <- chord_dat(data = circ, genes = dat, process = ego2$Term[1:6])

GOChord(chord, space = 0.02, gene.order = 'logFC', gene.space = 0.25, gene.size = 5)
