# Supplementary material for: Gene expression analysis in endometriosis: Immunopathology insights, transcription factors and therapeutic targets
Source: Front Immunol. 2022 Nov 30;13:1037504. doi: 10.3389/fimmu.2022.1037504 (PMC9748153; doi:10.3389/fimmu.2022.1037504)
Supplement: Supplementary file 1 [file DataSheet_1.zip › Raw data and code/Figure7/GSE11691/ROC curve codes for 5 TFs.docx]

library(tidyverse)

library(pROC)

library(ggplot2)

library(reshape2)

library(rstatix)

# data <- dat

data <- read.table("~/file.txt", header = T)

data$outcome <- factor(data$outcome, levels = c("group1", "group2"))

head(data)

# outcome a b c

# 1 group1 1.585855 1.1742805 2.674788

# 2 group1 2.205293 0.8619279 2.003079

# 3 group1 2.199554 2.3158722 1.281605

# 4 group1 1.241118 1.5746377 1.866428

# 5 group1 2.016992 1.9533336 1.847221

# 6 group1 2.391271 1.0891951 2.149648

data2 <- gather(data, key = "x", value = "value", -outcome)

data3 <- data2 %>%

group_by(outcome, x) %>%

get_summary_stats(value)

data3

# outcome x variable n min max median q1 q3 iqr mad mean sd se ci

# 1 group1 a value 40 0.586 2.44 1.45 1.12 1.94 0.822 0.631 1.51 0.555 0.088 0.177

# 2 group1 b value 40 0.556 2.50 1.8 1.14 2.21 1.07 0.777 1.65 0.619 0.098 0.198

# 3 group1 c value 40 1.05 2.95 2.07 1.77 2.44 0.665 0.495 2.04 0.527 0.083 0.169

# 4 group2 a value 32 0.129 1.99 0.996 0.623 1.50 0.878 0.633 0.998 0.554 0.098 0.2

# 5 group2 b value 32 0.022 1.96 0.779 0.325 1.18 0.86 0.624 0.843 0.572 0.101 0.206

# 6 group2 c value 32 0.03 1.89 0.923 0.421 1.39 0.969 0.706 0.932 0.546 0.097 0.197

roc1 <- roc(response = data$outcome, predictor = data$a)

# Call:

# roc.default(response = data$outcome, predictor = data$a)

#

# Data: data$a in 40 controls (data$outcome group1) > 32 cases (data$outcome group2).

# Area under the curve: 0.7328

roc2 <- roc(response = data$outcome, predictor = data$b)

# Call:

# roc.default(response = data$outcome, predictor = data$b)

#

# Data: data$b in 40 controls (data$outcome group1) > 32 cases (data$outcome group2).

# Area under the curve: 0.8234

roc3 <- roc(response = data$outcome, predictor = data$c)

# Call:

# roc.default(response = data$outcome, predictor = data$c)

#

# Data: data$c in 40 controls (data$outcome group1) > 32 cases (data$outcome group2).

# Area under the curve: 0.9242

ci.auc(roc1)

# 95% CI: 0.6171-0.8485 (DeLong)

ci.auc(roc2)

# 95% CI: 0.7303-0.9165 (DeLong)

ci.auc(roc3)

# 95% CI: 0.8679-0.9805 (DeLong)

coords(roc1, x = "best", ret="all")

# threshold specificity sensitivity accuracy tn tp fn fp npv ppv fdr fpr tpr tnr fnr

# threshold 1.115538 0.75 0.625 0.6944444 30 20 12 10 0.7142857 0.6666667 0.3333333 0.25 0.625 0.75 0.375

# 1-specificity 1-sensitivity 1-accuracy 1-npv 1-ppv precision recall youden closest.topleft

# threshold 0.25 0.375 0.3055556 0.2857143 0.3333333 0.6666667 0.625 1.375 0.203125

roc.test(roc1, roc2, reuse.auc=FALSE, method = "delong")

# DeLong's test for two correlated ROC curves

#

# data: roc1 and roc2

# Z = -1.2406, p-value = 0.2148

# alternative hypothesis: true difference in AUC is not equal to 0

# sample estimates:

# AUC of roc1 AUC of roc2

# 0.7328125 0.8234375

plot.roc(roc1)

data1 <- data.frame(group = "a",

x = 1-roc1$specificities,

y = roc1$sensitivities)

data1 <- data1[order(data1$x, data1$y),]

tmp <- data.frame(group = "b",

x = 1-roc2$specificities,

y = roc2$sensitivities)

tmp <- tmp[order(tmp$x, tmp$y),]

data1 <- rbind(data1, tmp)

data1$group <- factor(data1$group, levels = c("a", "b"))

ggplot() +

geom_line(data = data1, aes(x = x, y = y, colour = group)) +

labs(x = "1-Specificity (FPR)", y = "Sensitivity (TPR)")
