# Supplementary material for: Gene expression analysis in endometriosis: Immunopathology insights, transcription factors and therapeutic targets
Source: Front Immunol. 2022 Nov 30;13:1037504. doi: 10.3389/fimmu.2022.1037504 (PMC9748153; doi:10.3389/fimmu.2022.1037504)
Supplement: Supplementary file 3 [file DataSheet_3.pdf]

Supplementary Material

Supplementary Figures

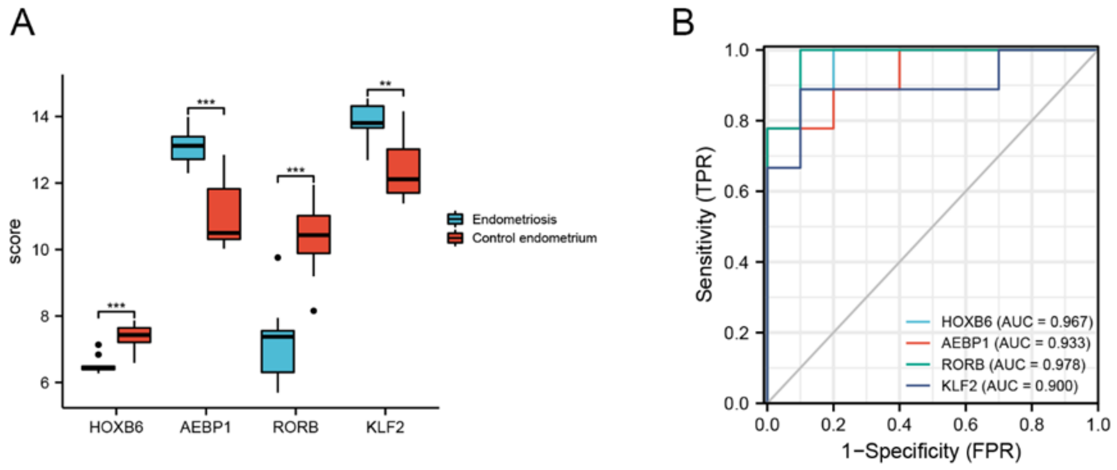

Supplementary Figure 1 | The expression (A) and ROC curves (B) for AEBP1, HOXB6, KLF2, and RORB in the GSE23339 dataset.

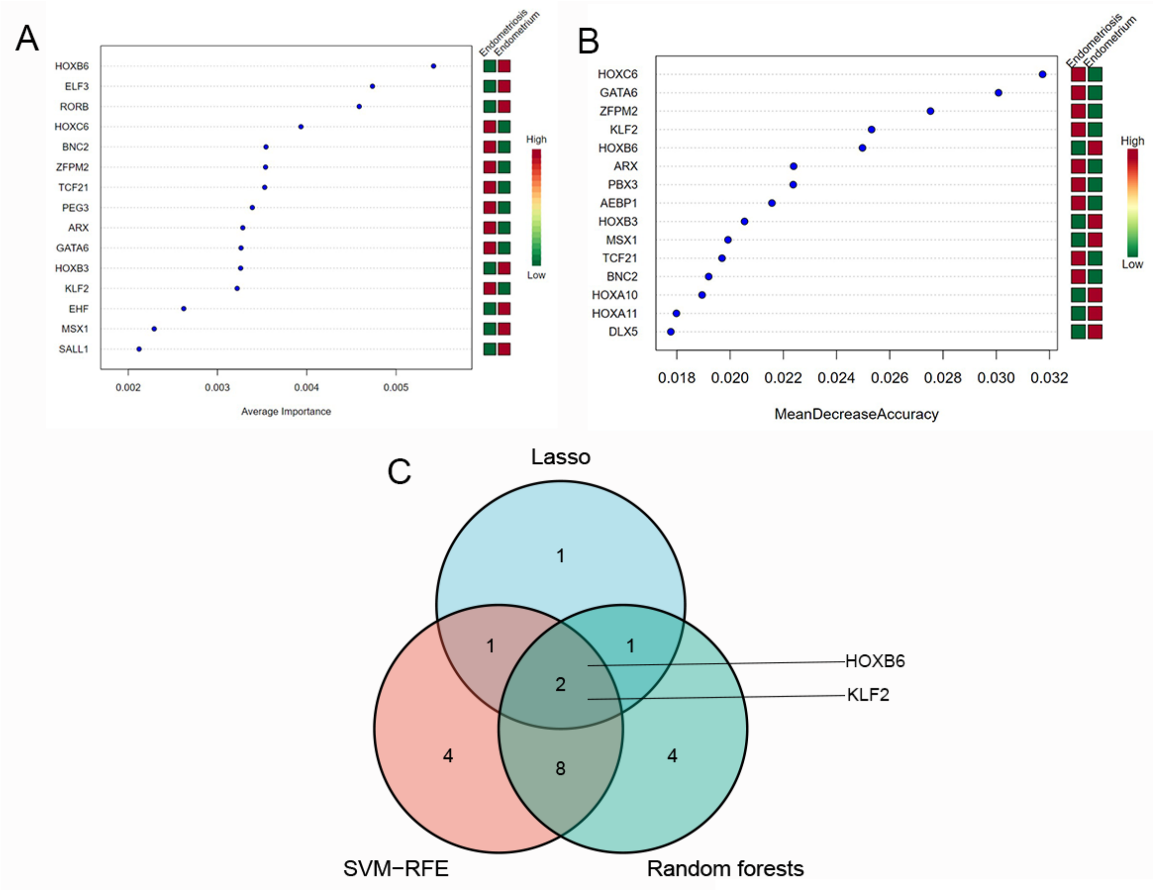

Supplementary Figure 2 | Multiple machine learning models identified key TFs (KLF2, HOXB6) for GSE7305. (A) The genes were selected based on RFE-SVMs algorithm. (B) Random forest model was employed to pick genes. (C) Intersection of genes generated from Lasso, RFE-SVMs, and Random forest model.
